# Supplementary material for: Maternal Infection and Adverse Pregnancy Outcomes among Pregnant Travellers: Results of the International Zika Virus in Pregnancy Registry
Source: Viruses. 2021 Feb 22;13(2):341. doi: 10.3390/v13020341 (PMC7926842; doi:10.3390/v13020341)
Supplement: Supplementary file 1 [file viruses-13-00341-s001.pdf]

Supplementary Material

		Major signs	Minor signs
**<u>Prenatal</u>**			
**Ultrasound**			
	**Cerebral**	Ventriculomegaly<br>Abnormal gyration pattern<br>Increased cisterna magna > 8mm<br>Vermian dysgenesis<br>Agenesis/dysgenesis of corpus callosum	Calcifications<br>Hypoplasia of the basal ganglia
	**Extracerebral**	Fetal hydrops<br>Arthrogryposis	IUGR < P10<br>Ascites, subcutaneous edema<br>ocular anomalies
**<u>Postnatal</u>**			
**Clinical signs**		Hypertonia<br>Swallowing disorder<br>HC < 3SD<br>Arthrogryposis<br>Macular anomalies<br>Abnormal cerebral MRI<br>Abnormal cerebral ultrasound with the exception of periventricular hemorrhagic	jaundice<br>Hepatomegaly<br>HC < 2SD<br>Abnormal audiology<br>Anterior segment anomalies
**Biological parameters**			Hb < 140 g/l<br>Tc < 100 G/L

Scheme S1. Criteria used to diagnose the severity of congenital Zika virus syndrome. Major signs were attributed three points, while minor signs were attributed one point. Foetuses/neonates were considered asymptomatic/pauci-symptomatic with a score ≤1, symptomatic with a score ≥2, and severely affected with a score ≥3.

	**Pregnant travellers**				**Pregnant residents**			
	**Recent maternal ZIKV infection** n=36		**Negative maternal ZIKV infection** n=108		**Recent maternal ZIKV infection** n=309		**Negative maternal ZIKV infection** n=412	
	n (%)	95% CI	n (%)	95% CI	n (%)	95% CI	n (%)	95% CI
**Fetal/neonatal testing**								
**Known**	23 (63.9)	47.6-77.5	6 (5.6)	2.6-11.6	293 (94.8)	91.7-96.8	340 (82.5)	78.6-85.9
**Positive**	**5 (21.7)**	**9.7-41.0**	**0 (0.0)**	n.a.	**76 (24.6)**	**20.1-29.7**	**1 (0.2)**	**0.0-1.4**
Asymptomatic	2 (40.0)	n.a.	n.a. n.a.	n.a.	33 (43.4)	32.9-54.6	1 (100.0)	n.a.
Severe adverse pregnancy outcomes	3 (60.0)	n.a.	n.a. n.a.	n.a.	28 (36.8)	26.9-48.1	0 (0.0)	n.a.
**Negative**	**18 (78.3)**	**58.1-90.3**	**6 (100.0)**	**61.0-100.0**	**217 (70.2)**	**64.9-75.1**	**339 (82.3)**	**78.5-85.9**
Asymptomatic	18 (100)	83.4-100.0	6 (100.0)	n.a.	188 (86.6)	81.5-90.5	294 (86.7)	82.7-89.9
Severe adverse pregnancy outcomes	0 (0.0)	0.0-17.6	0 (0.0)	n.a.	11 (5.1)	2.9-8.8	19 (5.6)	3.6-8.6
**Unknown**	**13 (36.1)**	**22.5-52.4**	**102 (94.4)**	**88.4-97.4**	**16 (5.2)**	**3.2-8.2**	**72 (17.5)**	**13.9-21.2**
Asymptomatic	13 (100)	77.2-100.0	97 (95.1)	89.0-97.9	16 (100.0)	80.6-100.0	70 (97.2)	90.4-99.2
Severe adverse pregnancy outcomes	0 (0.0)	0-22.8	4 (3.9)	1.5-9.7	0 (0.0)	0.0-19.4	2 (2.8)	0.8-9.6

Scheme S2. Adverse pregnancy outcomes according to results of foetal/neonatal ZIKV testing among pregnant travellers compared to pregnant residents.

Positive recent maternal ZIKV infection

	Case 1	Case 2	Case 3
**Outcome**			
Outcome	Severe cerebral anomalies (IUGR ventriculomegaly, cerebral calcifications)<br>Liveborn 37 WG; 1810g, HC 26 cm	Severe cerebral anomalies (ventriculomegaly, abnormal corpus callosum, calcifications, abnormal gyration)<br>Liveborn 37 WG, 2540g HC 30cm	Termination of pregnancy 14 WG for severe cerebral anomalies (ventriculomegaly, bilateral fetal neck collection)
GA at first anomaly seen	26 WG	19WG	14 WG
**Maternal characteristics**			
Maternal age	30 y.o.	41 y.o.	29 y.o.
Gestity/ Parity	4/1	4/1	4/3
Maternal country	North America	Europe	North America
Maternal comorbidities	None	None	None
Maternal serologies	Toxo neg; CMV neg; VZV ? ; HSV pos; Rub pos ; PVB19 neg ; Syph neg ; HIV neg; HCV ? ; HBV ?	Toxo neg; CMV ?; VZV ?; HSV ?; Rub pos;PVB19?; HIV neg; HCV neg; HBV neg	Toxo ?; CMV ? ; VZV ? ; HSV ? ; Rub ? ; HIV ? ; HCV ? ; HBV ?
DS screening	Normal 12 WG	?	?
**Fetal/neonatal investigations**			
Amniocentesis	not performed	19 WG	not performed
Fetal ZIKV testing	not performed	PCR pos	PCR pos
Placenta ZIKV testing	not performed	PCR neg	PCR pos
Neonatal ZIKV testing	5d - PCR neg; IgM pos	not performed	n.a.
Neonatal TORCH testing	CMV neg	CMV neg	n.a.
Genetic testing	?	?	not performed
Screening for metabolic disorders	normal	normal	not performed
**Maternal exposition**			
Region of travel	South America	South America	Caribbean Islands
Timing of travel	During the epidemic	During the epidemic	During the epidemic
Length of stay	> 2 weeks - < 3 weeks	> 4 weeks	?
Use of mosquitoes' repellent	?	?	?
Timing of infection	1T	1T (6 WG)	1T (9WG)
maternal symptoms	no	yes	yes
Maternal ZIKV diagnosis	IgM pos, Blood NAAT neg 37 WG	Blood NAAT pos at 12 WG	Blood NAAT pos at 12 WG; IgM pos at 13WG

Negative recent maternal infection

	Case 1	Case 2	Case 3	Case 4
**Outcome**				
Outcome	TOP 34WG for severe cerebral anomalies (abnormal gyration, ventriculomegaly) hepatomegaly, dysmorphism	IUGR, hypospadias, oligohydramnios, small thick placenta, polydactyly<br>Preterm delivery 27 WG, C-section, 720g, HIV 33cm HC	Hydrops fetalis and choledochal cyst<br>Preterm birth 33WG, C-section, 2180g, 33cm HC,	Isolated macular anomalies<br>Vaginal delivery 40 WG, 2970g, HC 33cm
GA at first anomaly seen	21 WG	19 WG	18 WG	at birth
**Maternal characteristics**				
Maternal age	35 y.o.	43 y.o.	37 y.o.	31 y.o.
Gravida/Parida	2/1	3/0	1/0	1/0
Maternal Origin	North America	North America	Europe	Europe
Maternal comorbidities	none	none	none	none
Maternal serologies	Toxo -, CMV -, VZV ?, HSV -, PVB-19 ?, Rub +, Syph -, HIV -, HCV ?, HBV -	oxo ?, CMV ?, VZV ?, HSV ?, Rub +, PVB-19 ?. Syph -, HIV -, HCV ?, HBV	Toxo +, CMV +, VZV +, HSV ?, Rub +, PVB-19 ?, Syph -, HIV -, HCV -, HBV -	Toxo -, CMV?, VZV ?, HSV ?, Rub ?, PVB-19 ?, Syph ?, HIV -, HCV -, HBV -
DS screening	Normal NIPT 20 WG	Abnormal 1/6 , Normal NIPT	Normal	
**Fetal/neonatal investigations**				
Amniocentesis	34 WG	not performed	not performed	not performed
Fetal ZIKV testing	not performed	not performed	not performed	not performed
Placenta ZIKV testing	not performed	not performed	not performed	
Neonatal ZIKV testing	not performed	not performed	not performed	Blood, urine, saliva and breast milk negative
Neonatal TORCH testing	?	not performed	not performed	not performed
Genetic testing	Pathogenic 4q21 deletion	3q21 deletion; unknown clinical significance	not performed	not performed
Screening for metabolic disorders	?	normal	not performed	not performed
**Maternal exposition**				
Region of travel	South America	Caribbean Islands	Africa	South America
Timing of travel	Outside the epidemic period	Outside the epidemic period	Outside the epidemic period	Outside the epidemic period
Length of stay	< 2 weeks	< 2 weeks	> 2 weeks - < 3 weeks	> 4 weeks
Use of mosquitoes' repellent	?	no	yes	?
Timing of exposition	1T	Prior to pregnancy	1T	prior to pregnancy
maternal symptoms	no	no	no	no
Maternal ZIKV diagnosis	IgM neg at 28 WG	IgM neg at 17 WG	Urine NAAT neg at 32 WG	Urine and Blood NAAT neg, IgM negative, IgG positive, PRNT positive at 10 WG,

Scheme S3. Description of pregnant travellers presenting with severe adverse pregnancy outcomes. Abbreviations: HC, Head circumference; NAAT, Nucleic acids amplification test; Neg, negative; Pos, Positive; T, trimester; WG, weeks' gestation; y.o.; years old; ZIKV, Zika virus
